# Should the biofilm mode of life be taken into consideration for microbial biocontrol agents?

**DOI:** 10.1111/1751-7915.12693

**Published:** 2017-02-16

**Authors:** Caroline Pandin, Dominique Le Coq, Alexis Canette, Stéphane Aymerich, Romain Briandet

**Affiliations:** ^1^ Micalis InstituteINRAAgroParisTechUniversité Paris‐Saclay78350Jouy‐en‐JosasFrance; ^2^ Micalis InstituteINRAAgroParisTechCNRSUniversité Paris‐Saclay78350Jouy‐en‐JosasFrance

## Abstract

Almost one‐third of crop yields are lost every year due to microbial alterations and diseases. The main control strategy to limit these losses is the use of an array of chemicals active against spoilage and unwanted pathogenic microorganisms. Their massive use has led to extensive environmental pollution, human poisoning and a variety of diseases. An emerging alternative to this chemical approach is the use of microbial biocontrol agents. Biopesticides have been used with success in several fields, but a better understanding of their mode of action is necessary to better control their activity and increase their use. Very few studies have considered that biofilms are the preferred mode of life of microorganisms in the target agricultural biotopes. Increasing evidence shows that the spatial organization of microbial communities on crop surfaces may drive important bioprotection mechanisms. The aim of this review is to summarize the evidence of biofilm formation by biocontrol agents on crops and discuss how this surface‐associated mode of life may influence their biology and interactions with other microorganisms and the host and, finally, their overall beneficial activity.

## Introduction

Approximately 30% of crop yields are lost every year worldwide, mostly due to diseases caused by pests, weeds or pathogenic microorganisms (Teng and Krupa, 1980; Teng, 1987; Oerke, 1999, 2006; Savary *et al*., 2012). The microbiological control of agricultural products along the food chain is still mainly ensured by the extensive use of chemical pesticides, preservatives and synthetic drugs (Horrigan *et al*., 2002). Environmental pollution and associated human diseases caused by this excessive use of chemicals during last century has led many agencies and governments worldwide to support an alternative route, where agriculture can be productive and economically viable, while still addressing societal and environmental concerns (Anonymous, 1999; Hazell and Wood, 2008; Aktar *et al*., 2009). Biological protection strategies are used and encouraged from farm to forks to prevent pathogen contaminations and livestock or crop diseases (Pal and McSpadden Gardener, 2006; Sundh and Melin, 2010; Jordan *et al*., 2014). Biological control, or ‘biocontrol’, consists in the removal of the harmful activity of one organism via one or more organisms or natural products extracted from microorganisms, plants, animals or minerals (Pal and McSpadden Gardener, 2006).

**Figure 1:**
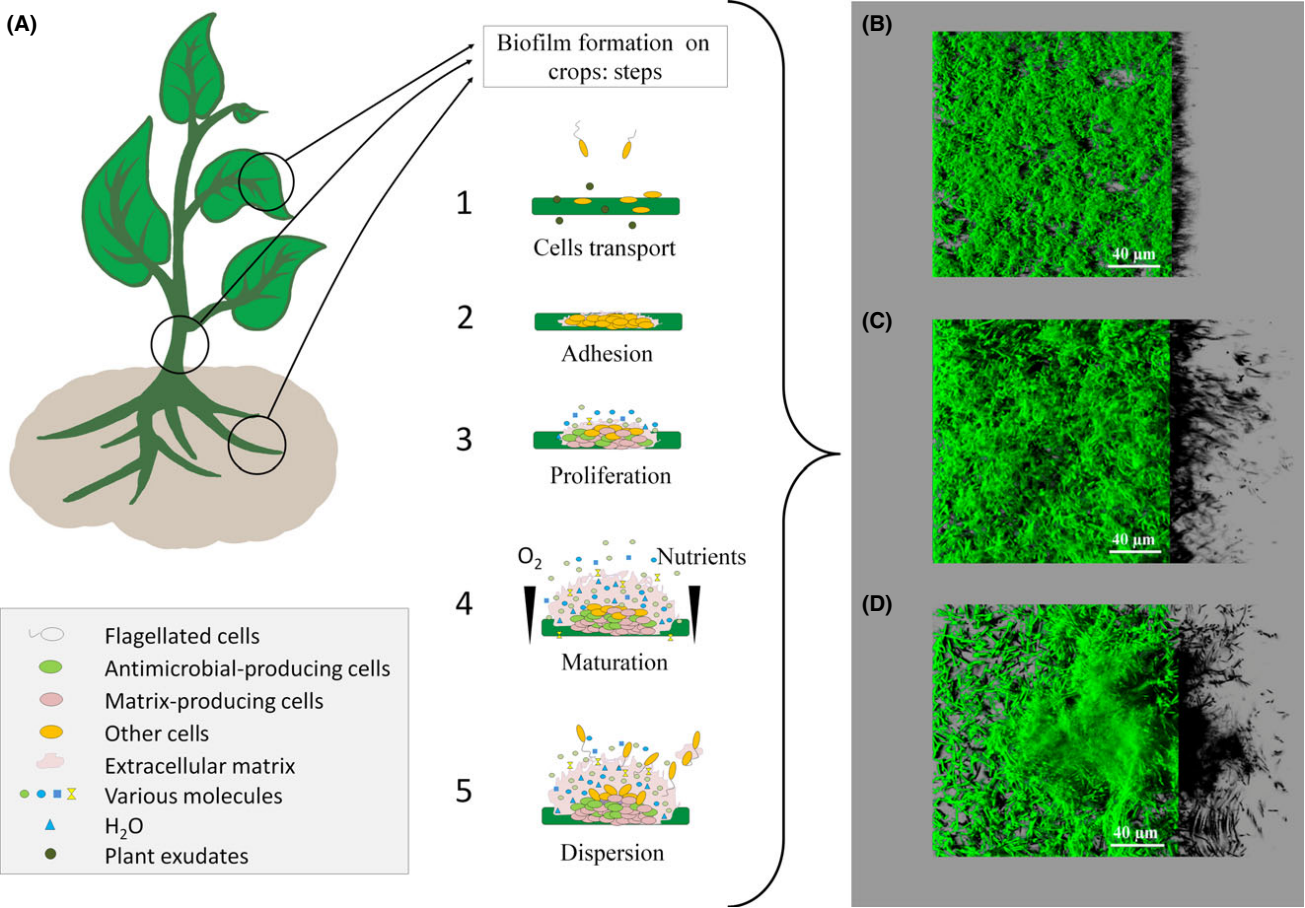
Biofilm formation on crops and *in vitro*: (A): On crops: The first step involves deposition on the substratum (1) followed by adhesion (2) to the support through cell wall decorations and extracellular appendages. Once attached, a proliferation phase (3) and the diversification of cell types initiate the spatial organization of the biostructure, leading to biofilm maturation (4). Biofilm ageing or environmental conditions unfavourable for the maintenance of the biofilm results in regulated dispersion of the biofilm (5), disseminating free cells and cell clusters that will start a new biofilm cycle on a new surface. B–D. *In vitro*: Structural diversity of three biocontrol agents as observed *in vitro* (24 h of axenic culture in microplates at 25°C) by confocal laser scanning microscopy (Leica SP8); (B) *Bacillus amyloliquefaciens* FZB42 expressing a green fluorescent protein (GFP), forming flat undifferentiated architecture, (C) *Bacillus amyloliquefaciens* SQR9 expressing a GFP and (D) *Bacillus subtilis* QST 713 (labelled in green with syto 9, Invitrogen, France) forming differentiated 3D biostructures.

**Figure 2:**
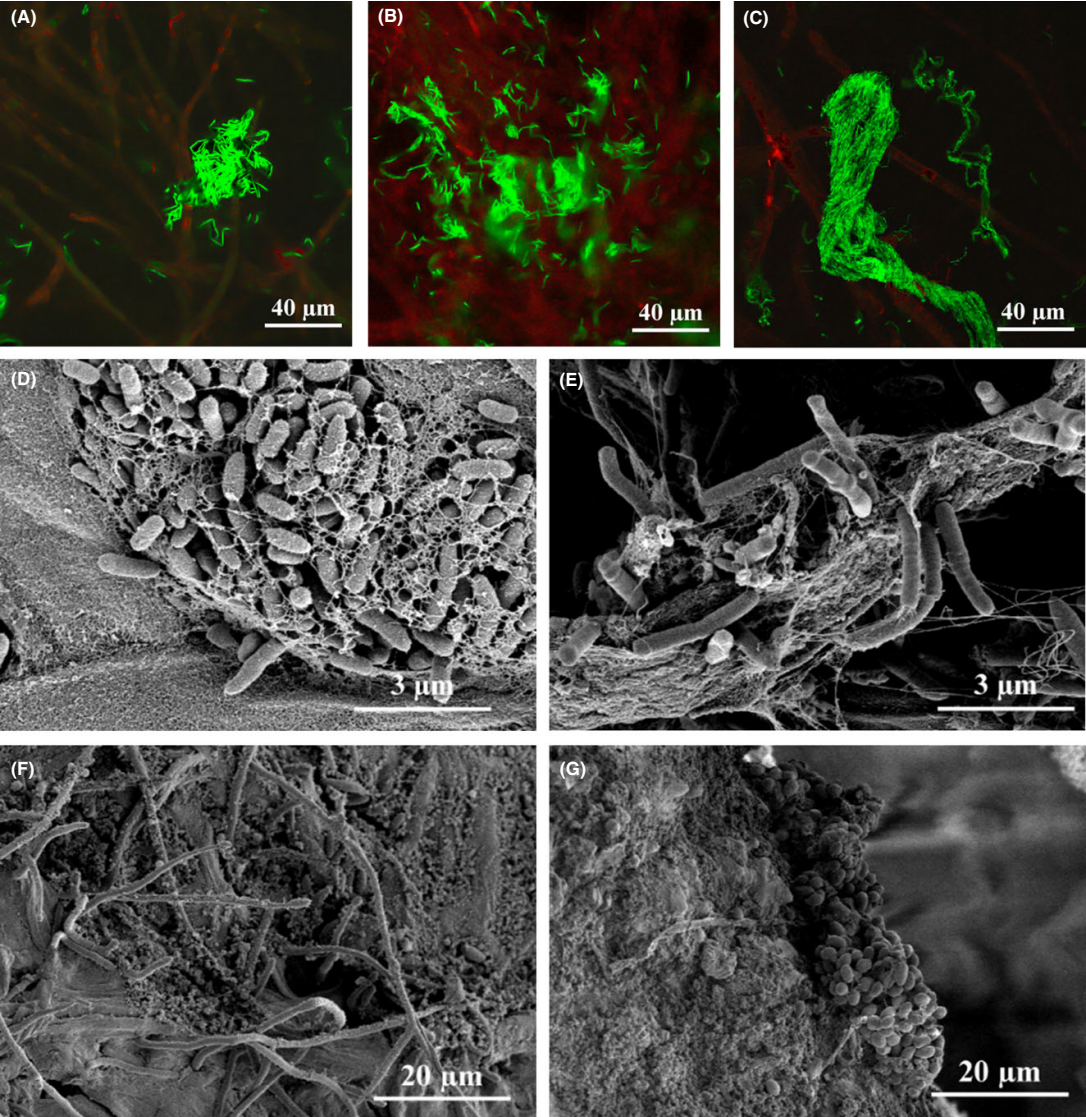
Microbial biofilms on the carpophore and culture compost of *Agaricus bisporus*. A–C. Confocal laser scanning microscopy of *Agaricus bisporus* carpophore (red autofluorescent hyphae), harbouring *Bacillus amyloliquefaciens* FZB42 expressing GFP and forming (A) clusters, (B) biofilm features and (C) bundles. *Agaricus bisporus* carpophores were immersed under axenic conditions in TSB (Tryptone Soy Broth, Sigma‐Aldrich, France) inoculated with *Bacillus amyloliquefaciens* FZB42 (GFP tagged) and incubated for 48 h at 17°C. Observations were performed using a Leica SP8 (Leica Microsystems, Danaher, Germany). D–G. Scanning electron microscopy of natural biofilms formed on *Agaricus bisporus* carpophore and compost protected with *Bacillus subtilis* QST 713, a biocontrol agent used at the French Mushroom Centre (Distré, France). Samples were fixed in 0.10 M cacodylate buffer containing 2.5% (v/v) glutaraldehyde (pH 7.4) and post‐fixed in 1% osmium tetroxide. Samples were then dehydrated with increasing concentrations of ethanol at room temperature (50–100%). After drying, samples were mounted on grids, sputter‐coated in argon plasma with platinum (Polaron SC7640, Elexience, France) and observed using a FE‐SEM S4500 (Hitachi, Japan). (D) *Pseudomonas*‐like bacteria with extracellular material, (E) *Bacillus*‐like bacteria, (F) fungi hyphae with extracellular material, (G) bacterial microcolony.

The relationship between survival, persistence and virulence of pathogenic microorganisms with their biofilm mode of life have been clearly established since the early 1980s (Costerton *et al*., 1978; Lam *et al*., 1980). According to the National Institute of Health, 80% of human infections involves microbial biofilms (NIH, 2002). Biofilm‐associated infections have also been reported in agricultural settings, e.g., in crops and animal diseases (Davey and O'toole, 2000; Prigent‐Combaret *et al*., 2012; Li *et al*., 2015). Indeed, the sessile mode is the preferential lifestyle of microorganisms, regardless of their biotope (Davey and O'toole, 2000; Morris and Monier, 2003). A biofilm can be described as a spatially structured community of microorganisms, generally embedded in an extracellular matrix, and adhering to a living or inert surface (Costerton *et al*., 1999; O'Toole *et al*., 2000). Biofilm formation is generally favoured in harsh environmental conditions, such as low nutritive or toxic media (Rendueles and Ghigo, 2015) and most bacteria can form biofilms in various environments (Morris and Monier, 2003; Aparna and Yadav, 2008). *Staphylococcus aureus* and *Pseudomonas aeruginosa* are two opportunistic pathogenic bacteria that cause a diverse set of diseases and are the most highly used model bacteria for biofilm studies. They can colonize the human nasopharynx and form biofilms when specific environmental conditions are met, causing invasive diseases, such as chronic pneumonia. These infections are difficult to treat because of the persistence of biofilms and their high resistance to antimicrobials (Blanchette and Orihuela, 2012; Ding *et al*., 2016a). Bacteria can colonize and form biofilms on stems, leaves and the rhizosphere of plants, as well as soil particles, mushrooms or organic compost (Figs 1A and 2) (Ramey *et al*., 2004; Prigent‐Combaret *et al*., 2012). For example, *Dickeya dadantii*, the causal agent of soft rot disease in a wide range of plant species, can colonize and form biofilms on chicory leaves, causing disease due to the production of degradative enzymes (Prigent‐Combaret *et al*., 2012; Pandin *et al*., 2016).

Although less explored, the formation of biofilms by moulds, yeast and algae, alone or in combination, in a variety of biotopes has also been reported (Morris and Monier, 2003; Aparna and Yadav, 2008; Zarnowski *et al*., 2014; He *et al*., 2016; Rajendran and Hu, 2016; Sheppard and Howell, 2016). *Aspergillus fumigatus,* a human pathogen, is a filamentous fungus that can form structured biofilms. The cohesive cement of the fungal biostructure is a polymeric extracellular matrix that protects the hyphae from the host immune system, similar to bacterial biofilms (Breitenbach *et al*., 2016; Mitchell *et al*., 2016; Sheppard and Howell, 2016; Shirazi *et al*., 2016). *Fusarium oxysporum* f. sp*. cucumerinum*, the pathogen responsible for cucumber Fusarium wilt, can also grow inter‐ and intracellularly, allowing the rapid colonization of the plant and biofilm formation (Li *et al*., 2015). Until recently, efforts in biofilm research have focused mainly on the medical field and essentially towards their eradication. With the emergence of biocontrol in agriculture, many microbiological products have been developed and are used in fields (Borriss, 2015). The main way of action of most of these commercial products is the antagonistic effect of antimicrobial molecules secreted by the biocontrol agent (Chowdhury *et al*., 2015; Mora *et al*., 2015). However, recent research in this field has made it possible to consider other major biological processes, including biofilm formation of biocontrol agents in crops (Bais *et al*., 2004; Bogino *et al*., 2013; De la Fuente *et al*., 2013).

## The formation of biofilms by microbial biocontrol agents

### Evidence of biofilm formation on crops by biocontrol agents

There is ongoing research to identify new biocontrol agents from environmental isolates and numerous biocontrol products have been developed and put on the agricultural market, mostly in Europe and North America (Borriss, 2015). Various products are in use and are effective on a wide range of plants. These include biofungicides, bactericides and biofertilizers based on *Bacillus subtilis* QST 713 or *Bacillus amyloliquefaciens* FZB42 (Borriss, 2015). These biocontrol products have an antagonistic effect towards unwanted microbes due to their secretion of antimicrobials, such as surfactin, fengycin or iturin (Ongena *et al*., 2005; Ongena and Jacques, 2008; Cawoy *et al*., 2014, 2015; Saravanakumar *et al*., 2016). However, their precise mechanisms of action in fields are still unknown. Few studies have focused on the determinants of effective bioprotection. The surface colonization step and biofilm formation by biocontrol agents are highlighted in the publications cited in Table**** 1. These reports demonstrate that many biocontrol agents can form biofilms on crops and in the rhizosphere. It has also been shown that biofilm formation by biopesticides can be stimulated by plant root exudates (Espinosa‐Urgel *et al*., 2002; Timmusk *et al*., 2005; Haggag and Timmusk, 2008; Khezri *et al*., 2011; Chen *et al*., 2013; Sang and Kim, 2014; Zhang *et al*., 2015), or by exposure of the microorganisms to antimicrobial products or stress (Bais *et al*., 2004; Selin *et al*., 2010; Fan *et al*., 2011; Xu *et al*., 2014; Chi *et al*., 2015; Wu *et al*., 2015; Zhou *et al*., 2016), but only a few studies have focused on biocontrol mechanisms that may be related to the properties of the mature biofilm itself, rather than the secretion of antimicrobials. *Bacillus* are ubiquitous spore forming bacteria predominantly found in soil. They are frequently used as biocontrol agents because they can sporulate and be stored for long periods (Branda *et al*., 2004; Borriss, 2015). *Bacillus amyloliquefaciens* FZB42 forms biofilms with little spatial organization *in vitro* (Fig. 1B), but exhibits a strong swarming capacity allowing a rapid surface colonization. For example, this strain can form biofilms on the fruiting body of *Agaricus bisporus* by forming bacterial clusters surrounded by extracellular matrix in contact with the mycelium of the carpophore (Fig. 2A and B), as well as cell bundles (Fig. 2C). Fan *et al*. (2011) reported the induction of biofilm formation of *B. amyloliquefaciens* FZB42 by root exudates of maize and surfactin. Similarly, surfactin triggers biofilm formation by *B. subtilis* UMAF6614 on the melon phylloplane (Zeriouh *et al*., 2014). Root exudate of cucumber also drives the chemotaxis of *Bacillus amyloliquefaciens* SQR9 and induces the production of bacillomycin D that triggers biofilm formation in the rhizosphere (Xu *et al*., 2014). Similarly, stem lesions of rice induce the production of GltB, leading to the production of bacillomycin L and surfactin, both involved in the biofilm formation of *B. subtilis* Bs916 (Zhou *et al*., 2016). Other biocontrol agents, such as endophytes, can also form biofilms. For example, some bacteria of the genus *Paenibacillus* form biofilms in wheat seeds and protect them from the invasion of *Fusarium graminearum* (Díaz Herrera *et al*., 2016).

**Table 1:** Biocontrol agent reported to form biofilms and the described associated biocontrol mechanisms

Biocontrol strain	Host/Location	Biofilm induction	Biocontrol mechanism	References
*Bacillus atrophaeus 176s*	Lettuce, sugar beet, tomato	Surfactin triggers biofilm formation	Induced systemic resistance (ISR) antimicrobial‐producing biofilm (fengycin, surfactin)	(Aleti *et al*., 2016)
*Bacillus subtilis*	Wheat seeds	Root exudates, death or lysis of cortex cells	Biofilm formation, antimicrobial, volatile compounds decrease mycelial growth	(Khezri *et al*., 2011)
*Bacillus subtilis* 3610	Tomato roots	Root exudates induce matrix	Antimicrobial‐producing biofilm (surfactin)	(Chen *et al*., 2013)
*Bacillus subtilis* 6051	*Arabidopsis thaliana*	Surfactin triggers biofilm formation	Antimicrobial‐producing biofilm (surfactin)	(Bais *et al*., 2004)
*Bacillus subtilis* Bs916	Rice stem	Stem lesions induce GltB production triggering bacillomycin L and, surfactin production involved in biofilm formation	Antimicrobial‐producing biofilm (fengycin)	(Zhou *et al*., 2016)
*Bacillus subtilis* UMAF6614	Melon phylloplane	Surfactin triggers biofilm formation	Antimicrobial‐producing biofilm (bacillomycin, fengycin)	(Zeriouh *et al*., 2014)
*Bacillus amyloliquefaciens* SQR9	Cucumber roots	Root exudates induce chemotaxis and enhance bacillomycin D production	Antimicrobial‐producing biofilm (bacillomycin)	(Xu *et al*., 2014)
*Bacillus amyloliquefaciens* SQR9	Maize roots	Root exudates induce the expression of genes related to extracellular matrix production	Promote plant growth	(Zhang *et al*., 2015)
*Bacillus amyloliquefaciens* SQY 162	Tobacco roots	Pectin enhances surfactin production, increasing biofilm biomass	May trigger induced systemic resistance (ISR) antimicrobial‐producing biofilm (surfactin)	(Wu *et al*., 2015)
*Bacillus amyloliquefaciens* FZB42	Maize roots	Root exudates and surfactin trigger biofilm formation	Likely not linked with the production of antibiotic or biofilm formation	(Fan *et al*., 2011)
*Paenibacillus polymyxa*	*Arabidopsis thaliana*	Root exudates induce matrix synthesis	Niche exclusion and mechanical protection	(Timmusk *et al*., 2005)
*Paenibacillus polymyxa* A26	Wheat seeds	Not mentioned	Niche exclusion of pathogens	(Abd El Daim *et al*., 2015)
*Paenibacillus polymyxa* B5	*Arabidopsis thaliana*	Root exudates	Niche exclusion of pathogens	(Haggag and Timmusk, 2008)
*Pseudomonas corrugata* CCR04 and CCR80	Pepper roots	Root exudates	Competitive colonization, such as swimming and swarming activities, biofilm formation, antimicrobial activity	(Sang and Kim, 2014)
*Pseudomonas chlororaphis* PA23	Canola roots	Phenazine enhances biofilm formation	Antimicrobial‐producing biofilm (pyrrolnitrin)	(Selin *et al*., 2010)
*Pseudomonas putida* 06909	Citrus roots	*Phytophthora* exudates as attractants and growth substrates for bacteria	Biofilm formation and mycelial colonization of the pathogen *Phytophtora*	(Steddom *et al*., 2002; Ahn *et al*., 2007)
*Pseudomonas putida* KT2440	Corn roots *Arabidopsis thaliana*	Root exudates	Promote plant growth and induced systemic resistance (ISR)	(Espinosa‐Urgel *et al*., 2002; Matilla *et al*., 2010)
*Pichia kudriavzevii*	Pear fruit	Oxidative stress	Greater activation of the antioxidant system in the biofilm form	(Chi *et al*., 2015)
*Kloeckera apiculate*	Citrus fruit	Phenylethanol promotes filamentous adhesion and biofilm formation	Niche exclusion and mechanical protection	(Pu *et al*., 2014)

Another family of biocontrol agents consists of the Gram‐negative *Pseudomonas*, ubiquitous bacteria found in many plant rhizospheres (Table 1) (Espinosa‐Urgel *et al*., 2002; Steddom *et al*., 2002; Matilla *et al*., 2010; Selin *et al*., 2010). Biofilm formation by *Pseudomonas putida* 06909 on citrus roots is induced by exudates of the phytopathogen *Phytophthora parasitica. *The bacteria colonize the mycelium of the fungi by feeding on its exudates and then form a protective biofilm on the citrus roots, which prevents new growth of the pathogen (Steddom *et al*., 2002; Ahn *et al*., 2007).

Living in a biofilm profoundly alters microbial properties relative to the planktonic mode of life (Whiteley *et al*., 2001; Shemesh *et al*., 2007; Vlamakis *et al*., 2008, 2013; Bridier *et al*., 2011b). Ongoing research is currently deciphering the molecular mechanisms involved in biofilm formation and their repercussions on biocontrol efficacy.

### Molecular mechanisms involved in biofilm formation of biocontrol agents

Until recently, few studies in the biocontrol field have considered that the preferred lifestyle of microorganisms in the environment is the biofilm mode of life. The main features associated with biofilm formation are a diversification of cell types and increased tolerance to the fluctuation of environmental factors, boosting microbial persistence in the environment (Vlamakis *et al*., 2008, 2013; Flemming *et al*., 2016). Bacteria and fungi can form biofilms on crops (as illustrated by the cultivated mushroom microbiota in Fig. 2), and in both cases, biofilm formation is composed of five major steps described in Fig. 1A (Costerton *et al*., 1999; O'Toole *et al*., 2000; Bianciotto *et al*., 2001; Davies, 2003; Triveni *et al*., 2012; Vlamakis *et al*., 2013; Pu *et al*., 2014; Haagensen *et al*., 2015; Li *et al*., 2015; Gulati and Nobile, 2016; Sheppard and Howell, 2016). *Bacillus subtilis* is the most highly documented bacterial model currently used to study the regulatory molecular mechanisms that govern biofilm formation. One specificity of the biofilm mode of life is the diversification of cell types. The presence of several bacterial subpopulations within the biofilm of *B. subtilis* has been clearly demonstrated, suggesting the spatiotemporal regulation of gene expression within such 3D structures (Vlamakis *et al*., 2008, 2013). Matrix‐producing cells, surfactin‐producing cells, flagellated motile cells and sporulated cells coexist in the same community (Fig. 1A) and are spatially and temporally organized, differentially expressing specific sets of genes (Vlamakis *et al*., 2008; van Gestel *et al*., 2015; Mielich‐Süss and Lopez, 2015; Wang *et al*., 2016). Indeed, the combination of surfactin‐ and matrix‐producing cells enables the organization of cells into bundles (Fig. 2C). These interfacial microbial cables allow bacteria to visit surrounding spaces to increase the biofilm surface area for nutrient and oxygen intake (van Gestel *et al*., 2015). Several genes involved in the phenotypic heterogeneity have been identified and extensively analysed in this species. For example, *hag*, encoding a flagellar protein and expressed by a subpopulation of motile cells; *tasA, eps, blsA* expressed by matrix‐producing cells; *sfrA*, involved in the production of surfactin lipopeptide; *sigF*, involved in cell sporulation; *swr*, involved in swarming motility; and the *com* genes, involved in genetic competence (Kearns *et al*., 2004; Verhamme *et al*., 2007; López and Kolter, 2010; Vlamakis *et al*., 2013; van Gestel *et al*., 2015; Mielich‐Süss and Lopez, 2015). All these genes are directly or indirectly regulated by various regulators (e.g. Spo0A, DegU, ComA, SinI, SinR, AbrB), which can thus play a role in the regulation of plant bioprotection by *B. subtilis* (López and Kolter, 2010; Vlamakis *et al*., 2013; Cairns *et al*., 2014; Mielich‐Süss and Lopez, 2015; Romero *et al*., 2016). Indeed, a mutation in a gene coding for a positive regulator (e.g. SinI) will decrease plant colonization and protection by diminishing attachment of cells to the roots, while mutations in a gene coding for a repressor (e.g. SinR, AbrB) will increase plant protection by an increased numbers of root‐attached cells and the formation of hyper‐robust biofilms (Chen *et al*., 2013).

Major components of biofilm structure that ensure its cohesion are the extracellular polymeric substances (EPS) that are mostly composed of water and extracellular biopolymers (polysaccharides, proteins, DNA, lipids) (Flemming and Wingender, 2010). Many microbial EPS have a backbone composed of various biomolecules forming gels with various cohesive and viscoelastic properties. Trapping a high amount of water is important for microbial survival against desiccation on plant surfaces (Abdian and Zorreguieta, 2016). This organic slime also protects their inhabitants from the action of environmental pollutants and toxic compounds (Sutherland, 2001; Sheppard and Howell, 2016). Another important component of the biofilm structure are amyloid fibres formed by the protein TasA. These filaments bind cells together, leading to formation of complex structures in biofilms that can hold and concentrate molecules (e.g. quorum sensing signalling molecules), and may also form aggregates to defend cells within the biofilm (de Jong *et al*., 2009; Romero *et al*., 2010; Flemming *et al*., 2016).

Several studies have recently highlighted various physiological behaviours of *Bacillus* within biofilm communities, demonstrating the high level of complexity of their interactions (Mitri *et al*., 2011; Houry *et al*., 2012; Liu *et al*., 2015; Prindle *et al*., 2015; Flemming *et al*., 2016). Prindle *et al*. (2015) described a new function for ion channels in biofilms in which they conduct electrical signals *via* spatial propagation of potassium waves which depolarize adjoining cells and coordinate the state of the exterior and interior cells of the biofilm. In addition, Liu *et al*. (2015) discovered a ‘collective oscillation’ phenomenon involved in toxic chemical tolerance, based on metabolic codependency between exterior and interior cells of the biofilm, and consisting of cyclic pauses during biofilm growth which increase the availability of nutrients in the deepest layers. Houry *et al*. (2012) also demonstrated that motile bacilli, expressing a bactericide, can kill a heterologous biofilm population and then occupy the newly created space (Houry *et al*., 2012). Altogether, these cellular traits show the complexity of living associated with a surface in a spatially organized microbial community. They also give an overview of the protection that biofilms can provide to their inhabitants on plant surfaces. Those basic insights into biofilm development and interaction might pave our way towards various applications in the field of crop protection.

## Biofilm‐specific properties that should be considered in biocontrol mechanisms

**Figure 3:**
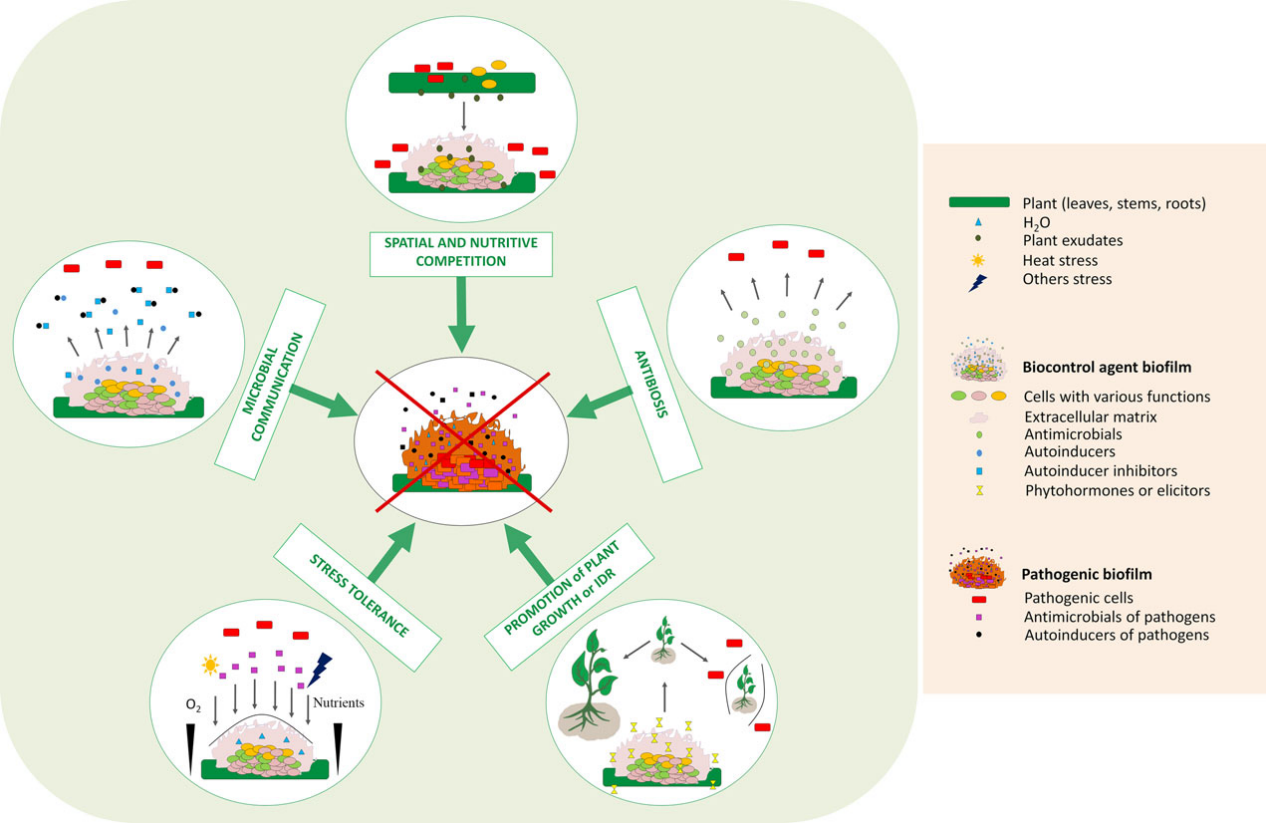
Proposed mechanisms of plant interactions with biocontrol agents and pathogenic strains. (IDR: induced disease resistance).

Only a few published studies have considered the possibility of interspecies and microbial–host interactions in spatially organized plurimicrobial biofilms involved in agricultural biocontrol (De la Fuente *et al*., 2013; Triveni *et al*., 2015) (Table 1). The biofilm‐associated properties to be considered can be divided into five classes (Fig. 3): (i) antagonism by niche exclusion orchestrated by spatial and nutritive competition (Timmusk *et al*., 2005; Haggag and Timmusk, 2008; Pu *et al*., 2014; Abd El Daim *et al*., 2015), (ii) microbial communication, e.g. cooperation/interference (Hogan *et al*., 2004; Audrain *et al*., 2015; Chen *et al*., 2015), (iii) production of antimicrobials by biofilm cells (Bais *et al*., 2004; Selin *et al*., 2010; Chen *et al*., 2013; Sang and Kim, 2014; Xu *et al*., 2014; Zeriouh *et al*., 2014; Wu *et al*., 2015; Zhou *et al*., 2016), (iv) stress tolerance (Timmusk *et al*., 2005; Harriott and Noverr, 2009; Pu *et al*., 2014) and (v) direct effects on plant physiology, e.g. the induction of plant defences (Wu *et al*., 2015) and/or stimulation of plant growth (Espinosa‐Urgel *et al*., 2002; Zhang *et al*., 2015). This new vision could significantly change our understanding of the interactions involved in biocontrol by considering them in terms of spatial/nutritive competition (Habimana *et al*., 2011), tolerance/resistance (Bridier *et al*., 2011a) or their physiology, as microorganisms in a biofilm differ greatly from their planktonic homologues (Stewart and Franklin, 2008). These local processes are described in the following sections, using illustrative examples from other fields, if they have not been explored yet in the microbial biocontrol area.

### Spatial and nutritive competition

The spatial organization of biocontrol agent biofilms on crop surfaces varies depending on their genetic potential and the environmental conditions. For example, the biofilms of *Bacillus* biocontrol agents display wide architectural diversity between strains. *In vitro,* biofilms of *B. amyloliquefaciens* SQR9 and *B. subtilis* QST 713 exhibit the classical thick and highly organized 3D structure of bacilli (Fig. 1C and D). In contrast, *B. amyloliquefaciens* FZB42 forms only thin structures of a few cell layers (Fig. 1B). However, this strain outcompetes the other two due to its swarming activity, leading to rapid coverage of the entire surface. This ability to rapidly colonize a niche (Fig. 3) has been described previously as a potential biocontrol mechanism and could be called upon for the strain *B. amyloliquefaciens* FZB42 (Timmusk *et al*., 2005; Haggag and Timmusk, 2008; Fan *et al*., 2011; Abd El Daim *et al*., 2015). For *Paenibacillus polymyxa,* root exudates of plants induce invasive root colonization and biofilm formation that invades sites that could be potentially occupied by pathogens, thus preventing them from settling onto the surface by forming a protective biofilm (Timmusk *et al*., 2005; Haggag and Timmusk, 2008; Abd El Daim *et al*., 2015). In an organized, 3D community, nutrients may be consumed faster than they can diffuse throughout the matrix (Breugelmans *et al*., 2008; Stewart and Franklin, 2008). Growth and survival in such a dense community is frequently associated with spatial competition. Habimana *et al*. (2011) explained the inhibition of *Listeria monocytogenes* by *Lactococcus lactis* on surfaces by considering the 3D race between the two species. Using a simplified individual‐based model approach, they demonstrated that the differences in the growth parameters (lag phase and growth rate) of the two species could explain the observed inhibition of the pathogenic cells. *Lactococcus lactis* cells rapidly formed layers on the mixed community and completely saturated the interface in contact with the nutrient, limiting nutrient access to the pathogen. This example illustrates that part of a biofilm population can be starved within the bulk of the biostructure, even in a very rich environment. In addition, Liu *et al*. (2016) underlined that the specific interactions between species, such as strong or weak cooperation, exploitation or competition, contribute mostly to the spatial organization of biofilms, as these interactions create fitness effects in multispecies biofilms. Taking spatial organization and interspecies interactions within multispecies biofilms into account could increase our understanding of the interactions that take place in agrosystems that use biocontrol agents.

### Antibiosis

The production of secondary metabolites by selected organisms is one of the best described mechanisms of agricultural microbial biocontrol (Ongena *et al*., 2005; Ongena and Jacques, 2008; Khezri *et al*., 2011; Cawoy *et al*., 2014, 2015; Chen *et al*., 2015; Aleti *et al*., 2016; Raza *et al*., 2016; Saravanakumar *et al*., 2016). *Bacillus* genomes contain many genes involved in the production of secondary metabolites, recently compiled in an exhaustive classification of known and putative antimicrobial compounds (Zhao and Kuipers, 2016). Indeed, 4–5% of the genome of *B. subtilis* is allocated to the production of antibiotics and 8.5% of the genome of *B. amyloliquefaciens* FZB42 is allocated to the production of secondary metabolites with antimicrobial properties (Stein, 2005; Chen *et al*., 2009; Zhao and Kuipers, 2016). Many exhibit interesting antibacterial properties (e.g. difficidin), antifungal properties (e.g. bacillomycin D, fengycin and surfactin), or both (e.g. bacilysin) (Ongena and Jacques, 2008; Chen *et al*., 2009; Guo *et al*., 2014; Guo *et al*., 2015; Chowdhury *et al*., 2015; Luo *et al*., 2015; Kröber *et al*., 2016). Most of the studies that have analysed the profile of antimicrobial production have relied on experiments using planktonic laboratory cultures. However, in *B. amyloliquefaciens* FZB42, the genes involved in bacilysin synthesis are overexpressed in biofilms, suggesting that the bacteria have a stronger antagonistic effect in their sessile mode of life (Fig. 3) (Kröber *et al*., 2016). Similarly, in *B. subtilis*, the regulator NtdR controls the expression of the *ntdABC* operon, encoding enzymes involved in the biosynthesis of the antibiotic kanosamine (Inaoka *et al*., 2004; Vetter *et al*., 2013). A global transcriptomic study that compared gene expression of *B. subtilis* in various modes of life showed that this operon is strongly overexpressed in biofilms (Nicolas *et al*., 2012), suggesting the possible involvement of kanosamine in interspecies interactions in plurimicrobial biofilms. Volatile organic compounds (VOCs) can also trigger antimicrobial activity (Khezri *et al*., 2011; Audrain *et al*., 2015; Raza *et al*., 2016). Raza *et al*. (2016) demonstrated that VOCs of *B. amyloliquefaciens* SQR9 inhibited the growth of *Ralstonia solanacearum* on agar medium or in soil. Altogether, these studies show that secondary metabolites with antimicrobial activity can be overproduced (or simply produced) in the biofilm lifestyle, improving antagonistic biocontrol activity. The presence of EPS or amyloid fibres in biofilms can also locally concentrate these molecules and prevent their dilution into the ambient aqueous environment, and thus presumably increase the virulence of biocontrol agents against pathogens in agrosystems (Bianciotto *et al*., 2001; Romero *et al*., 2010; Xu *et al*., 2014; Flemming *et al*., 2016). Previous studies highlighted effects of antimicrobials secreted by one producer on crop protection. Santhanam *et al*. (2015) have also shown that in certain cases, a consortium of different antimicrobial producers is required for optimal plant bioprotection.

### Microbial communication

Biofilms are dense, spatially organized communities of microorganisms with extensive forms of social life. They can use specific signalling molecules (autoinducers) that allow them to sense and communicate with the local surrounding populations (Fuqua *et al*., 1994). This quorum sensing (QS) is involved in various biological processes, such as swarming, stress tolerance (pH, antimicrobials, etc.), the production of secondary metabolites, horizontal gene transfer, colonization, biofilm maturation and the synthesis of virulence factors (Fuqua *et al*., 1994; Von Bodman *et al*., 2003). These signalling pathways are widely used in bacteria–bacteria and bacteria–eukaryote associations to regulate and coordinate their interactions. For example, *N*‐acylhomoserine lactones (AHL) in Gram‐negative bacteria, oligopeptides in Gram‐positive bacteria and gamma‐butyrolactones in species of the genus *Streptomyces* are autoinducers (Danhorn and Fuqua, 2007). In *Pseudomonas aeruginosa*, QS controls the expression of many bacterial functions. The LasI‐LasR QS system, with the autoinducer synthase LasI and the signal receptor LasR, is involved in biofilm maturation and the organization of its 3D structure. A *lasI* mutant can initiate biofilm formation but is unable to form a mature biofilm, suggesting that LasI is involved in the late stages of biofilm development (Davies *et al*., 1998; De Kievit *et al*., 2001). Kavanaugh and Horswill (2016) demonstrated that the *Staphylococci* QS system, *agr*, is involved in biofilm disruption and dispersal.

In the field of biocontrol, it was shown that the protective activity of *Pseudomonas fluorescens* 2P24 on wheat was mainly controlled by the PcoI‐PcoR QS system that governs biofilm formation, and not directly by the production of antimicrobial metabolites (Wei and Zhang, 2006). Such social behaviour has been shown to also govern intermicrobial and interkingdom interactions, such as communication interference represented in Fig. 3 or cooperative communication (Zhang and Dong, 2004; Kalia, 2013). For example, *P. aeruginosa* secretes 3‐oxo‐C12‐HSL that affects the growth of *C. albicans* hyphae and inhibits its biofilm formation (Hogan *et al*., 2004). Amyloid fibres of the matrix form aggregates that can act as QS inhibitors by binding QS signalling molecules, and thus locally concentrate these molecules that can reach a 1000‐fold higher concentration in the matrix than in planktonic cell environments (Charlton *et al*., 2000; Hense *et al*., 2007; Romero *et al*., 2010; Flemming *et al*., 2016). Other types of molecules can quench or degrade QS signalling molecules of another species (Zhang and Dong, 2004). Indeed, AHL‐lactonase of *Bacillus thuringiensis* hinders the accumulation of AHL of *Erwinia carotovora*, thus decreasing the virulence of this bacterium on potatoes (Dong *et al*., 2004). In *Bacillus*, the lipopeptide surfactin, in addition to its antibiotic properties, can act like a signalling molecule to promote biofilm formation of the other relative *Bacillus* (López *et al*., 2009; Aleti *et al*., 2016). Volatile organic compounds emitted by prokaryotic and eukaryotic microbes are also part of their communication repertoire and can trigger global reprogramming of gene expression of their perceivers (Farag *et al*., 2013; Audrain *et al*., 2015; Raza *et al*., 2016). For example, acetic acid emitted by *B. subtilis* biofilms can promote biofilm formation of other physically separated *B. subtilis* cells and thus act as an important pathway of cell–cell communication (Audrain *et al*., 2015; Chen *et al*., 2015). These communication phenomena specific to biofilms, or amplified in biofilms, could be used to improve biocontrol in agrosystems.

### Stress tolerance: adaptability properties and matrix as a protective shield

Bacteria in biofilms exhibit specific physiologies associated with increased tolerance/resistance of the overall community to harsh conditions (Costerton *et al*., 1999; Whiteley *et al*., 2001; Shemesh *et al*., 2007; Bridier *et al*., 2011b). Physiological differences between sessile and planktonic cells are mostly related to differential patterns of gene expression associated with the two modes of life (Whiteley *et al*., 2001; Shemesh *et al*., 2007). Transcriptomic analysis of *Streptococcus mutans*, a bacterium associated with tooth decay, showed that 12% of the genome was differentially expressed in biofilm communities relative to their single‐cell homologues. The differentially expressed genes coding for known functions are involved in transport, signalling, metabolism, protein and antimicrobial synthesis (Shemesh *et al*., 2007). Mark *et al*. (2005) evaluated the influence of exudates of two varieties of sugar beets on the transcriptomic profile of *Pseudomonas aeruginosa* PAO1. They showed that the expression of 516 genes was altered in response to one exudates and 451 to the other, and 134 genes responded to both. They found that genes coding for the synthesis of alginate, a major component of the biofilm matrix, were upregulated. These results suggest that *P. aeruginosa* PAO1 forms a biofilm in response to sugar beet exudates. They also showed that the transcriptomic profile of *Pseudomonas aeruginosa* PAO1 in response to exudates is variety dependent. Similarly, Matilla *et al*. (2007) compared the transcriptome profiles of *Pseudomonas putida* KT2440 in the planktonic exponential growth phase, the planktonic stationary growth phase, the sessile form, in sand microcosms and in the rhizosphere. They showed that transcriptomic profile of the planktonic mode of life in the stationary growth phase was the most different from that of the rhizosphere, whereas that of the biofilm lifestyle was more comparable. Indeed, they found that the gene involved in the synthesis of alginate was upregulated in the rhizosphere (Matilla *et al*., 2007; : additional data file). These ‘omics’ studies confirm the presence of biofilm formation in the rhizosphere or in response to plant exudates. These techniques should be increasingly considered in the study of microbial interactions in agrosystems and extended to metagenomics, metaproteomics and metatranscriptomic approaches as successfully performed in other fields (Blottière *et al*., 2013; Kaul *et al*., 2016).

Other cellular variations can occur during biofilm formation. In the early stages, *Pseudomonas aeruginosa* shows enhanced genetic diversification. Resulting phenotypes vary from cells involved in accelerated biofilm formation to those with enhanced dispersion properties. In the first case, biofilms exhibited pronounced spatial differentiation leading to rough and wrinkled colonies on agar. In the second case, the biofilms showed little spatial organization resulting in small and flat colonies (Boles *et al*., 2004). These differences illustrate the genetic plasticity of cells within a biofilm that enables them to cope with harsh environmental conditions. Stewart and Franklin (2008) also reported the existence of nutrient and oxygen gradients within biofilms creating a stratification of local microenvironments associated with a diversification of cell physiologies (Fig. 3). Population heterogeneity can generate multiple regulatory pathways leading, for example, to the phenomenon of competence in a subpopulation of cells, which coupled with the spatial proximity, facilitates horizontal gene transfer between biofilm cells. This can include the acquisition of plasmids carrying antimicrobial resistance genes (Witte, 2000; Abraham, 2011; Kung and Almeida, 2014; Liu *et al*., 2016). This diversification of cell types in biofilms strongly suggests that the biofilm lifestyle of biocontrol agents enables them to better adapt to, and resist, the hostile conditions encountered in agrosystems (the so‐called insurance effects in Boles *et al*., 2004) than their planktonic counterparts.

Biofilms are ubiquitous and subject to harsh conditions, such as the presence of antimicrobials or desiccation. Stewart (2015) recently performed a meta‐analysis of the literature from which he proposed that biofilm tolerance to antimicrobials depends neither on the size or chemistry of the antimicrobials nor the composition of the microbial biofilm or the material to which it adheres (Stewart, 2015). Based on his analysis, biofilm‐associated tolerance is primarily related to the nature and composition of the biofilm matrix. Indeed, the composition of the matrix creates various meshes within the biofilm leading to a diffusion–reaction limitation that can reduce antimicrobial penetration and local biodisponibility (Fig. 3) (Stewart *et al*., 2001; Stewart and Franklin, 2008; Flemming and Wingender, 2010; Bridier *et al*., 2011b; Stewart, 2015). Stewart (2015) also stressed that the presence of the matrix and the associated 3D organization renders slow‐growth populations less sensitive to certain stresses than their planktonic counterparts.

The matrix also plays a central role in interspecies and interkingdom interactions. *Staphylococcus aureus* and *Candida albicans* are often associated in human diseases, where they form a polymicrobial biofilm (Harriott and Noverr, 2009; Lindsay and Hogan, 2014). This association allows *S. aureus* to resist vancomycin, an antibiotic that is usually efficient against the planktonic form of *S. aureus*. The biofilm of *C. albicans* serves as the backbone of *S. aureus* microcolonies that form on their surface with the matrix of *C. albicans* covering and protecting the cells of *S. aureus* from the action of the antibiotic (Harriott and Noverr, 2009). Other reports have described the protection of *Staphylococcus epidermidis* by *C. albicans* (Adam *et al*., 2002) or of *S. aureus* by *B. subtilis* (Sanchez‐Vizuete *et al*., 2015). In the latter case, the authors identified a single gene of the *B. subtilis* NDmed whose disruption suppressed the protective effect. This gene (*ypqP)* is likely involved in the production of matrix exopolysaccharides (Sanchez‐Vizuete *et al*., 2015). Non‐polysaccharidic components of the matrix can also contribute to the matrix shield; the amphiphilic protein BlsA produced by *B. subtilis* prevents the penetration of biocides by forming a hydrophobic ‘raincoat’ layer at the biofilm–air interface (Epstein *et al*., 2011; Kobayashi and Iwano, 2012). Biocontrol agents likely benefit from this protective shield on crop surfaces to avoid invasion by aggressive detrimental flora and protect crops.

### The direct response of crops to biocontrol agents

Plants can develop natural defence systems against pathogenic microorganisms during their interactions with their environment (biotic and abiotic) (Pieterse and Wees, 2015). Several induced diseases resistance (IDR) mechanisms have been described, including induced systemic resistance (ISR) that is an innate defence mechanism of the plant (Choudhary and Johri, 2009; Pieterse and Wees, 2015) elicited by various environmental stimuli, such as VOCs and QS signalling molecules (Farag *et al*., 2006; Choudhary and Johri, 2009; Matilla *et al*., 2010; Wu *et al*., 2015; Aleti *et al*., 2016). Various stimuli, such as VOCs, QS signals and phytohormones produced and concentrated in the biofilm matrix, can stimulate plant growth, analogous to ISR (Fig. 3) (Espinosa‐Urgel *et al*., 2002; Farag *et al*., 2006; Han *et al*., 2006; Spaepen, 2015; Zhang *et al*., 2015; Díaz Herrera *et al*., 2016; Ding *et al*., 2016b). Thus, VOCs, originating from various sources, can induce ISR and plant growth (Yi *et al*., 2009; Farag *et al*., 2013; Audrain *et al*., 2015; Raza *et al*., 2016; Sharifi and Ryu, 2016). These host responses can also be induced by products coming from plant growth‐promoting rhizobacteria that have already colonized the root surface, or endophyte colonization of its host (Whipps, 2001; Farag *et al*., 2006; Borriss, 2015; Díaz Herrera *et al*., 2016). The plant growth‐promoting rhizobacteria *B. subtilis* GB03 and *B. amyloliquefaciens* IN937a can produce 2,3‐butanediol and acetoin on plant roots and promote both plant growth and ISR by eliciting these phenomena (Ryu *et al*., 2003, 2004; Farag *et al*., 2006). Han *et al*. (2006) also showed that the surface area of tobacco leaves increased when they were exposed to 2,3‐butanediol secreted by *Pseudomonas chlororaphis* O6 or exposed to the strain itself, even without physical contact. Phytohormones (auxins, cytokinins, gibberellins, abscisic acid and ethylene) are elicitors, which can be produced by bacteria and play a role in promoting plant growth (Spaepen, 2015; Zhang *et al*., 2015). The auxin, indole‐3‐acetic acid, is produced by *B. amyloliquefaciens* SQR9 and *B. amyloliquefaciens* FZB42 biofilms and promotes the growth of maize and *Lemna minor* (Chen *et al*., 2007; Idris *et al*., 2007; Zhang *et al*., 2015). Endophytes can promote growth of wheat and protect it from *Fusarium graminearum* (Díaz Herrera *et al*., 2016).

## Perspectives for sustainable agroecological approaches

Biocontrol mechanisms triggered by biological control agents in agriculture are not yet well understood, and even unknown in certain cases. A single biocontrol agent can use a combination of various biocontrol processes, best described for the strain *B. amyloliquefaciens* FZB42. The use of this bacilli can lead to antagonism, spatial and nutritional competition, antimicrobial production, the stimulation of plant growth and the induction of plant resistance (Timmusk *et al*., 2005; Haggag and Timmusk, 2008; Babalola, 2010; Fan *et al*., 2011; Xu *et al*., 2011; Kröber *et al*., 2014; Chowdhury *et al*., 2015; Kröber *et al*., 2016; Abd El Daim *et al*., 2015). The biofilm mode of life is still poorly taken into account in biocontrol, although it clearly plays a role in agrosystems and governs some of the observed beneficial effects. It would be informative, in the near future, to include phenotypic screening of the ability of strains to form biofilms as a rapid selection criterion of biocontrol agents. Several high‐throughput screening tests that could be used for this application are described in the literature (Azeredo *et al*., 2016). Better genetic knowledge of the various cell functions in biofilms will also open doors to selection criteria based on the presence of specific genes involved in important and specific biocontrol functions (Kaul *et al*., 2016).

Invoking biofilm formation as a determinant of biocontrol efficacy could be a new attractive strategy to better control its beneficial effects. This could be achieved, for example, using natural biofilm promoting molecules that trigger the biocontrol agent QS response. In the case of *B. subtilis*, this could be surfactin, VOCs, specific microbial exopolysaccharides or crop extracts (Bais *et al*., 2004; Chen *et al*., 2013; Chen *et al*., 2015; Audrain *et al*., 2015; Zhou *et al*., 2016). This effect could also be obtained by adding a second strain (or more) with the ability to stimulate biofilm formation by the initial biocontrol agent, for example through VOC synthesis (Filoche *et al*., 2004; Audrain *et al*., 2015; Chen *et al*., 2015; Figueiredo *et al*., 2016). Attention should be paid, in this case, to select strains without antagonistic activity against each other or the beneficial action of the biocontrol agent, as previously reported for certain cocktails in the literature (Xu *et al*., 2011). The benefit of the biofilm mode of life for biocontrol agents could also be obtained using dedicated formulations, as suggested in other areas; for example, the development of new formulas grown as biofilms to orally administer probiotics (e.g. beads of agar or alginates) is under consideration (Rieu *et al*., 2014). Similarly, a system using bacteria‐containing polymersomes, which permits rapid biofilm growth, has been developed for bioremediation to reduce the toxicity of environmental selenium contamination (Barlow *et al*., 2016).

Increasing evidence, based on available data from the agrosystem and biofilm fields, strongly suggests that the combination of features associated with the 3D biofilm mode of life should be considered when evaluating the performance of biocontrol organisms.

## Conflict of interest

None declared.
